# Association between urinary zinc excretion and isoflavone-metabolizing enterotypes among Japanese females: a cross-sectional study

**DOI:** 10.1265/ehpm.23-00148

**Published:** 2023-10-27

**Authors:** Tomoko Fujitani, Zhaoqing Lyu, Mariko Harada Sassa, Kouji H. Harada

**Affiliations:** Department of Health and Environmental Sciences, Kyoto University Graduate School of Medicine, Kyoto 606-8501, Japan

**Keywords:** Enterotype, Isoflavone, Metabolism, Zinc, Japanese

## Abstract

**Background:**

Zinc absorption and competition among gut bacteria have been reported in animal studies. Thus, gut bacteria may modify zinc availability in humans. Metabolism of intestinal bacteria is known to be necessary for the activation of several phytoconstituents in the body. For example, equol, a typical substance of soybean isoflavone, is produced by intestinal bacteria metabolizing daidzein and the enterotype is one of distinct ones among Japanese population. The difference in the intestinal microflora can modify the bioavailability of zinc. In this study, we examined urinary zinc concentrations in adult female equol producers (EQPs).

**Methods:**

Urine samples from women participating in health examinations in Miyagi, Okinawa, Kyoto, Kochi, and Hokkaido prefectures were used; from total 17,484 samples, approximately 25 samples were randomly selected for each age group from 30 to 60 years per region (subsample: n = 520), and 520 samples with available urinary zinc concentration (determined by flame atomic absorption analysis) and enterobacterial type were analyzed. EQP was defined as log(equol/daidzein) ≥ −1.42, and urinary concentrations were corrected for creatinine concentration. Urinary zinc concentrations were compared by Student’s *t*-test and multiple regression analyses.

**Results:**

The geometric mean urinary zinc concentration (µg/g-Cr) was lower in EQP than in non-EQP (p = 0.0136 by *t*-test after logarithm transformation). On the other hand, there was no correlation between urinary zinc concentration with daidzein (r = −0.0495, P = 0.436) and equol concentrations (r = −0.0721, P = 0.256). There was a significant negative association between urinary zinc concentration and EQP (β = −0.392, P = 0.0311) after adjusting with other potential confounding variables, such as daidzein intake.

**Conclusions:**

The results suggest that gut bacteria that produce equol are involved in the metabolism of zinc. Based on previous studies, the bacteria that affect the metabolism of both substances are thought to be *Enterococcus*. Future studies are expected to identify specific intestinal bacteria for zinc availability and understand individual differences in the effects of micronutrients.

**Supplementary information:**

The online version contains supplementary material available at https://doi.org/10.1265/ehpm.23-00148.

## Introduction

Zinc is an essential micronutrient that plays a critical role in numerous physiological processes in the human body [1]. As a cofactor for over 300 enzymes, zinc is involved in a wide range of metabolic pathways, including protein synthesis, DNA synthesis and repair, cell division, and immune function [2]. Zinc is also essential for proper growth and development, and is particularly important during pregnancy and infancy when rapid growth and development are occurring [3, 4].

Zinc is obtained through the diet and is absorbed in the small intestine. It is then transported to various tissues, including the gut, where it plays a critical role in maintaining the integrity of the intestinal barrier and modulating immune function. Studies have also shown that zinc can influence the composition and function of the intestinal microbiota [5], although the exact mechanisms underlying this relationship remain unclear.

The human gut is a complex ecosystem of microorganisms, collectively known as the intestinal microflora, which plays a crucial role in maintaining gut homeostasis and overall health. Recent studies have shown that dietary factors can modulate the composition and function of the intestinal microbiota [6], leading to alterations in host metabolism and immune response. One such dietary factor that has gained attention in recent years is zinc [7]. The absorption of zinc occurs primarily in the small intestine, where it is transported across the intestinal epithelium by specific transporters, such as ZIP4 and ZnT1 [8]. The expression and activity of these transporters can be influenced by the gut microbiota. For example, ZIP8, one of zinc transporters is induced under inflammatory stimuli [9]. Dysbiosis, or an imbalance in the composition of the intestinal microflora, can also impact zinc absorption. Studies have shown that dysbiosis is associated with reduced nutrient absorption and increased fecal excretion. Zinc competition among the microbiota can occur in the gastrointestinal tract. Zinc uptake by a high-affinity ZnuABC transporter is detected in *Campylobacter jejuni*. It promotes survival and growth of *C. jejuni* [10]. Given the importance of both zinc and the intestinal microbiota in maintaining gut homeostasis and overall health, understanding the interplay between these two factors is of significant interest.

Soybeans are frequently consumed in Asian countries and are rich in isoflavones. Isoflavones are known to be metabolized by intestinal bacteria [11]. The main soy isoflavone, daidzein, is metabolized and absorbed by intestinal bacteria, and those who have intestinal bacteria capable of metabolizing to equol are defined as equol producers (EQP) [10]. EQPs have been reported to be associated with a lower risk of developing hormone-derived cancers, fewer menopausal symptoms, and less osteoporosis, among other health outcomes [12]. Several bacteria have been reported to be in intestinal microflora in EQPs, and their metabolism requires multiple bacteria [13]. Actually, among Japanese females, 39% can metabolize daidzein to equol [14]. Thus, the EQP status is one of the distinct enterotypes. The type of bacteria in the gut may modify the availability of trace elements such as zinc.

This study examined the potential association between urinary zinc concentrations and EQP status in Japanese adult females.

## Methods

### Study participants

In the original study, 17,484 female participants were recruited during medical check-ups in 11 prefectures in Japan between 2000 and 2009 [15]. Subjects were collected during the annual health checkups at different workplaces. Therefore, adult workers were included. There were no particular criteria for exclusion at the time of collection. Those without sufficient urine volumes were excluded because they could not be analyzed for isoflavones and elements. Written informed consent was obtained from all participants before sample collection. Urine samples were collected at a time of no menstruation and were stored at −30 °C for later analysis at the Kyoto University Human Specimen Bank [16].

For this study, 520 non-pregnant women from five prefectures recruited between November 2000 and December 2001 were extracted from the specimen bank [17]. A total of 100–110 samples per prefecture from the original participant group were randomly selected and analyzed for urinary isoflavone levels and selected elements. The protocol of this study was approved by the Ethics Committee of Kyoto University Graduate School of Medicine and Faculty of Medicine and Hospital (Latest approval number R1478-10 on May 31st, 2022, ‘Human exposure monitoring and risk assessment’).

All participants were of Asian ethnicity. Age and body mass index (BMI) of participants were obtained from health records. The participants’ parity, smoking habits, present and past disease histories and menstrual status were recorded using a self-report questionnaire. There were two people with pancreatic disease among the subjects.

### Measurement of urinary polyphenols and elements

Polyphenols in the urine were analyzed using a gas chromatography-mass spectrometer (6890GC/5973MSD; Agilent Technologies Japan Ltd., Tokyo, Japan) as previously described by Liu et al. [14]. The measured elements were urinary zinc, calcium, magnesium, lead and cadmium as described in the previous report [15]. Zinc was analyzed by atomic absorption spectrophotometry (AAS). Calcium was analyzed by ortho-cresal phthalein complexone. Magnesium was analyzed by xylidyl blue. Lead and cadmium were analyzed by flameless AAS. Urinary creatinine concentration was assayed by using enzymatic method.

### Statistical analysis

Values under the detection limits were recorded as half of the detection limit. The urinary concentrations were normalized to urinary creatinine amounts (g-Cr). To enable a statistical analysis comparison, the urinary concentrations were transformed into common logarithmic values because the distributions were not normal. EQPs were defined according to the criteria proposed by Ideno et al. [18]: log(equol/daidzein) ≥ −1.42 indicates EQP status, and log(equol/daidzein) < −1.42 indicates non-EQP status.

Urinary zinc concentrations between EQP and non-EQP were compared by Student’s *t*-test. To adjust the potential confounders (age, BMI, etc.), we conducted multivariable regression analyses. Records with missing values were excluded from the main analyses while the missing values (smoking: 19, menstrual status: 13) were processed by single imputation methods (mode imputations) as additional analyses. JMP Pro (ver. 15; SAS Institute, Cary, NC) was used to perform these calculations. The alpha level for all tests was 0.05.

## Results

Table 1 shows urinary polyphenol and element concentrations separately for menstrual status, smoking status, BMI, and disease history. Their mean age was 49.2 yr old (standard deviation 10.1). 143 participants had disease histories but pancreatic diseases, which can modify zinc excretion, existed only in two participants. Menstrual status was associated only with urinary equol concentration (P = 0.0225), but not with zinc (P = 0.143) or daidzein concentration (P = 0.0567). The results showed that smoking status was not associated with urinary zinc concentration (P = 0.212). BMI was marginally associated with equol concentration (P = 0.0687), zinc concentration (P = 0.169), and daidzein concentration (P = 0.174).

**Table 1:** Characteristics of the study subjects and the urinary polyphenol and element concentrations.

		**equol** **(µmol/g-Cr)**	**daidzein** **(µmol/g-Cr)**	**trans resveratrol** **(nmol/g-Cr)**	**epicatechin** **(nmol/g-Cr)**
Total (N = 520)		0.324 (6.45)	6.61 (4.30)	24.6 (3.29)	41.1 (7.76)

Menstrual cycle					
Regular cycles	240 (47.3%)	0.250 (6.24)	5.48 (4.07)	18.9 (3.11)	31.4 (7.18)
Irregular cycles	43 (8.4%)	0.482 (7.26)	6.94 (4.32)	28.5 (2.23)	27.4 (5.98)
Menopause	187 (36.9%)	0.410 (6.46)	7.94 (4.56)	33.9 (3.41)	55.3 (8.55)
Experienced gynecological surgery	37 (7.3%)	0.333 (6.12)	7.99 (4.45)	23.4 (2.78)	95.2 (6.89)
Smoking habit					
Non-smoker	428 (85.4%)	0.355 (6.44)	6.58 (4.33)	24.7 (3.40)	41.8 (8.13)
Current smoker	58 (11.6%)	0.154 (5.19)	6.30 (3.80)	21.7 (3.12)	25.5 (4.88)
Ex-smoker	15 (3.0%)	0.367 (9.15)	4.13 (5.47)	34.3 (2.05)	58.4 (10.07)
BMI					
Underweight	84 (16.2%)	0.488 (7.10)	7.02 (4.13)	26.0 (4.49)	59.3 (8.50)
Normal	320 (61.5%)	0.288 (6.48)	6.54 (4.42)	25.1 (3.12)	36.2 (7.87)
Overweight and obesity	116 (22.3%)	0.332 (5.75)	6.53 (4.14)	22.6 (2.96)	44.6 (6.81)

Disease history					
None	377 (72.5%)	0.333 (6.61)	6.32 (4.17)	23.5 (3.21)	36.1 (7.30)
Current/past histories	143 (27.5%)	0.301 (6.07)	7.46 (4.63)	28.1 (3.48)	57.7 (8.76)

		**Zn** **(µmol/g-Cr)**	**Mg** **(mmol/g-Cr)**	**Ca** **(mmol/g-Cr)**	**Pb** **(nmol/g-Cr)**	**Cd** **(nmol/g-Cr)**
Total (N = 520)		4.19 (2.21)	2.37 (1.60)	2.46 (1.86)	6.64 (1.78)	10.3 (2.02)

Menstrual cycle						
Regular cycles	240 (47.3%)	4.38 (1.96)	2.10 (1.63)	1.97 (1.88)	5.60 (1.60)	8.06 (1.97)
Irregular cycles	43 (8.4%)	4.26 (2.00)	2.28 (1.57)	2.59 (2.04)	7.73 (1.94)	10.7 (1.88)
Menopause	187 (36.9%)	4.22 (2.31)	2.75 (1.43)	3.08 (1.67)	8.96 (1.82)	12.9 (1.94)
Experienced gynecological surgery	37 (7.3%)	3.15 (3.72)	2.41 (1.89)	2.91 (1.63)	6.88 (1.54)	14.7 (1.92)
Smoking habit						
Non-smoker	428 (85.4%)	4.05 (2.27)	2.38 (1.58)	2.53 (1.81)	6.69 (1.82)	10.2 (1.99)
Current smoker	58 (11.6%)	4.89 (1.80)	2.24 (1.61)	2.06 (2.09)	6.97 (1.53)	10.7 (1.96)
Ex-smoker	15 (3.0%)	4.59 (2.08)	2.37 (2.02)	2.46 (1.85)	3.69 (1.06)	9.06 (2.89)
BMI						
Underweight	84 (16.2%)	3.88 (2.43)	2.42 (1.54)	2.59 (1.79)	7.48 (1.59)	9.13 (2.07)
Normal	320 (61.5%)	4.41 (2.12)	2.35 (1.60)	2.42 (1.87)	6.87 (1.85)	10.6 (2.02)
Overweight and obesity	116 (22.3%)	3.83 (2.28)	2.40 (1.62)	2.46 (1.86)	5.52 (1.60)	10.5 (1.98)

Disease history						
None	377 (72.5%)	4.17 (2.06)	2.31 (1.57)	2.37 (1.89)	6.61 (1.81)	9.91 (2.01)
Current/past histories	143 (27.5%)	4.22 (2.60)	2.55 (1.64)	2.72 (1.73)	6.76 (1.66)	11.6 (2.01)

Concentrations were expressed as geometric means and geometric standard deviation.

In Table 2, urinary concentrations were compared between EQP and non-EQP. Urinary zinc levels were lower in EQP than in non-EQP (P = 0.0136). Urinary daidzein concentrations were higher in non-EQP than in EQP since the EQP can metabolize daidzein to equol. There was no difference in other elements and polyphenols between EQP and non-EQP. Since Ca and Mg share similar metabolic pathways in the kidney, we examined their balance (Ca/Mg ratio) by EQP status. Urinary Ca/Mg ratio was relatively lower in EQP than in non-EQP (P = 0.0648) (Table 2).

**Table 2:** Urinary concentrations of elements and polyphenols among equol producers and non-producers.

	**N**	**EQP**	**non-EQP**	**P value**
		**250 (48.0%)**	**270 (51.9%)**	
**Elements**				
Zn (µmol/g-Cr)	520	3.83 (2.33)	4.55 (2.09)	0.0136
Mg (mmol/g-Cr)	520	2.39 (1.56)	2.35 (1.63)	0.618
Ca (mmol/g-Cr)	520	2.39 (1.86)	2.53 (1.85)	0.302
Cd (nmol/g-Cr)	520	11.0 (1.96)	9.79 (2.06)	0.0629
Pb (nmol/g-Cr)	125	6.85 (1.82)	6.45 (1.73)	0.566
Ca to Mg ratio (mole basis)	520	1.88 (0.916)	2.03 (1.03)	0.0648

**Polyphenols**				
Daidzein (µmol/g-Cr)	520	3.29 (4.83)	12.6 (2.63)	<0.0001
Equol (µmol/g-Cr)	520	1.15 (6.53)	0.0998 (2.11)	<0.0001
Trans resveratrol (nmol/g-Cr)	520	23.5 (3.16)	25.7 (3.41)	0.388
Epicatechin (nmol/g-Cr)	520	41.6 (7.32)	40.6 (8.21)	0.897

Concentrations were expressed as geometric means and geometric standard deviation. g-Cr: grams creatinine; Cd: cadmium; Pb: lead; Mg: magnesium; Ca: calcium; Zn: zinc. P values indicate the results from two-tailed Student’s *t*-test between EQP and non-EQP groups. Log-transformed concentrations of urinary equol, daidzein, and creatinine, were used for this test.

Soybeans contain zinc and phytic acid as well as daidzein and modify zinc absorption [19], thus the association between EQP status and urinary zinc might be due to differences in soybean intake. To examine the possibility, correlations between urinary zinc and daidzein/equol concentrations were investigated in EQP (N = 250) (supplementary information, Table S1). There was no correlation between urinary zinc concentration with daidzein (r = −0.0495, P = 0.436) and equol concentrations (r = −0.0721, P = 0.256). Furthermore, to eliminate the potential confounding effects from demographic factors, analyses of covariance were performed (Table 3). There was a significant negative association between urinary zinc concentration and EQP (β = −0.392, P = 0.0311) after adjusting with other variables.

**Table 3:** Analysis of covariance of the association between log urinary Zn and EQP, including other variables.

**Dependent Variable: Log urinary Zn (µmol/gCr)**			

**Independent variables**	**β**	**95% CI**	**P**
BMI			
(normal weight)	0.0307	−0.0118–0.0732	0.157
(overweight and obese)	−0.00375	−0.0559–0.0484	0.888
Age (yr)	0.00188	−0.00386–0.00763	0.519
Log daidzein (µmol/gCr)	−0.0131	−0.0687–0.0425	0.644
EQP	−0.0392	−0.0748–−0.00357	0.0311
Smoking habit			
(current smoker)	0.0152	−0.0712–0.102	0.730
(ex-smoker)	0.0232	−0.0991–0.145	0.710
Menstrual status			
(irregular cycles)	0.0630	−0.0267–0.153	0.168
(menopause)	0.0227	−0.0477–0.0932	0.527
(experienced gynecological surgeries)	−0.157	−0.265–−0.0482	0.00474
Disease histories (current or past)	0.0247	−0.0142–0.0637	0.213

CI: confidence interval. Analysis of covariance was conducted for log urinary Zn as dependent variable with independent variables (EQP, log daidzein, age and smoking) (N = 491). Log daidzein, age, and smoking were included as covariables in addition to EQP in this analysis. BMI was coded as 1 = underweight, 2 = normal weight, 3 = overweight and obese. Equol status was coded as 1 = EQP, 2 = non-EQP. Tobacco use was coded as 1 = non-smoker, 2 = current smoker, and 3 = ex-smoker. Menstrual status was coded as 1 = regular cycles, 2 = irregular cycles, 3 = menopause, and 4 = experienced gynecological surgery. Disease history was coded as 1 = person with current or past disease histories, and 2 = person without disease histories. Category 1 was set as a reference. Model fitness: R^2^ = 0.0383.

Additional analyses were conducted to examine potential biases or confounding. We have performed a multivariable regression analysis in disease-free participants to eliminate the potential effects from drugs and treatments such as antibiotics, chemotherapy, and hormonal therapy. The results showed the same decreasing trend as in Table 3 (EQP, β = −0.0243, P = 0.214) (supplementary information, Table S2) while it was not statistical significant due to the limited statistical power. Furthermore, analyses by imputation of missing values showed similar association between urinary zinc concentration and EQP to the main analysis (β = −0.0359, P = 0.0391) (supplementary Table S3).

## Discussion

In this cross-sectional study, the association between urinary zinc concentration and EQP status was observed in Japanese females. Experience of gynecological surgeries was also associated with reduced urinary zinc concentrations while multivariable regression analysis showed an independent effect of EQP status on urinary zinc levels. Since zinc absorption does not fluctuate with medium- or short-term zinc administration [20], the difference in urinary zinc concentrations were not likely to be a result of short-term soybean consumption, daidzein, or equol. Indeed, there was no correlation among urinary zinc, daidzein and equol concentrations. Thus, intestinal microflora that is responsible for equol metabolisms might affect the bioavailability of zinc. On the other hand, there were no associations between EQP status and other elements and polyphenols (except for daidzein). In subgroup analyses, disease-free individuals showed a similar trend and thus effects of drugs such as antibiotics, chemotherapy, or hormonal therapy did not have significant effects (Table S2). Missing values were found in smoking and menstrual statuses but the association between urinary zinc and EQP remained significant in the imputed analysis (Table S3).

Ca/Mg ratio in urine was lower in EQP than in non-EQP. Previous studies have shown that the composition of the intestinal microflora changed in rats treated with Mg supplementation [21], and it is possible that the intestinal microflora for EQP might be involved in the metabolism of Mg or Ca. However, the association was not statistically significant and further study for confirmation should be required.

Although this study analyzed the association between EQP enterotypes and urinary zinc levels, the underlying mechanism remains unclear. However, two possibilities may be speculated. First, zinc could be taken up by the EQP-producing bacteria for their growth and metabolism, resulting in lower urinary zinc concentrations. For example, the metabolism of Fe was influenced by *Lactobacillus fermentum* [22]. The gut bacteria that express ZnuABC and ZupT transporters and compete with intestinal absorption of zinc [7]. In broilers, competition for zinc utilization between *Campylobacter jejuni* and the host has been observed [10]. In addition, zinc uptake by pathogens is regulated by the zinc uptake repressor (Zur) and also in *S. aureus*, the zinc efflux repressor (CzrA) was identified [23]. Equol-producing bacteria include *Lactococcus* 20-92, *Eggerthella*, *Lactococcus garvieae*, *Slackia isoflavoniconvertens*, and *Adlercreutzia equolifaciens* [24]. Overlapped microbes that metabolize daidzein and zinc are needed to be identified using metagenome analyses [25].

A second possibility is that bacterial zinc absorption might be inhibited in non-EQP. Upon sensing a bacterial infection, the immune systems sequester zinc and iron. The general strategy so-called nutritional immunity induces host proteins such as lactoferrin, calprotectin, and lipocalin-2 released from neutrophils and macrophages [26]. Those proteins bind to iron and zinc, and reduce their concentrations in tissues and in the intestinal tract [27, 28]. Therefore, it is possible that in non-EQP, the intestinal bacteria might be unable to take up zinc and as a result of the accelerated efflux from bacterial cells, zinc was absorbed by hosts and urinary concentrations were higher than in EQP [29]. Immunological characteristics of EQP enterotype should be investigated in future works.

There are several limitations. This is a cross-sectional study and therefore the relationship is only at a single point and causal relationship could not be determined. In addition, there might be potential biases while the background information of participants was limited. To eliminate the confounding from disease histories, subgroup analysis was conducted in disease-free participants and similar trend was found while other factors may exist. For example, lower albumin levels might be associated with decreases in trace elements including zinc [30]. In a previous study by Nagata et al. (2009), relationship between dietary intake and urinary equol concentration was examined showing that women who consumed more dairy products excreted less equol than those who did not [31]. Also, soybean intake is a potential factor for EQP and nutritional intake, but urinary concentrations of daidzein and equol were not associated with urinary zinc (Table S1). In the future, background factors such as lifestyle, biochemical data that can affect the intestinal microbiota should be included. In this study, enterotype was limited to EQP and association was found only with zinc, but the comprehensive analysis of microbiome will show the possible relationships between those metabolisms and enterotypes.

This study may imply the importance of intestinal microbiota in zinc bioavailability. External intake of zinc does not necessarily represent the actual internal levels of zinc. Biomonitoring of zinc using serum/urine or determination of enterotypes would improve the exposure assessment in epidemiological studies. In view of public health nutrition, the dietary intake recommendations such as the “Dietary Reference Intakes for Japanese” can consider these differences in bioavailability. Those recommendations provide estimated average requirements and recommended dietary allowance while in this study, it was found that some people, such as EQP, may less utilize zinc than other populations. By focusing on such individual differences, it may be possible to propose nutritional intakes suited to each individual as personalized and precision preventive medicine. In this study, EQP status rather than urinary isoflavone levels was associated with urinary zinc levels, suggesting that intestinal microflora may be a determinant. Numerous reports found that 20% to 40% of the population was EQP [32], so this result can be applicable to populations outside Japan with limited soy consumptions.

## Conclusion

The results suggested that the gut bacteria that produce equol might be involved in the bioavailability of zinc.

## Abbreviations

AAS: atomic absorption spectrophotometry
ANOVA: analysis of variance
BMI: body mass index
EQP: equol producer

## Supplementary information

**Additional file 1:** **Table S1.** Correlation between urinary zinc and daidzein and equol in EQP (N = 250). **Table S2.** Analysis of covariance of the association between log urinary Zn and EQP in participants without disease histories. **Table S3.** Association between log urinary Zn and EQP with imputations of missing values in smoking habit and menstrual status.

## Declarations

### Data availability statement

The data that support the findings of this study are available from the corresponding author upon reasonable request.
