# Supplementary material for: Association between urinary zinc excretion and isoflavone-metabolizing enterotypes among Japanese females: a cross-sectional study
Source: Environ Health Prev Med. 2023 Oct 27;28:63. doi: 10.1265/ehpm.23-00148 (PMC10613555; doi:10.1265/ehpm.23-00148)
Supplement: Supplementary file 1 — Additional file 1: Table S1. Correlation between urinary zinc and daidzein and equol in EQP (N = 250). Table S2. Analysis of covariance of the association between log urinary Zn and EQP in participants without disease histories. Table S3. Association between log urinary Zn and EQP with imputations of missing values in smoking habit and menstrual status. [file ehpm-28-063-s001.docx]

**Table S1.** Correlation between urinary zinc and daidzein and equol in EQP (N=250).

|  | Log daidzein (μmol/g-Cr) | Log equol (μmol/g-Cr) |
| --- | --- | --- |
| Log Zn (μmol/g-Cr) | r= -0.0495  (P= 0.436) | r=-0.0721  (P=0.256) |

**Table S2.** Analysis of covariance of the association between log urinary Zn and EQP in participants without disease histories.

| **Dependent variable: Log urinary Zn (μmol/gCr)** | | | | |  |
| --- | --- | --- | --- | --- | --- |
| **Independent variables** |  | β | 95% CI | P | |
| BMI |  |  |  |  | |
| (normal weight) |  | 0.0486 | 0.00321 – 0.0941 | 0.0359 | |
| (overweight and obese) |  | -0.0351 | -0.0903 – 0.0202 | 0.213 | |
| Age (yr) |  | -0.00228 | -0.00881 – 0.00425 | 0.492 | |
| Log daidzein (μg/gCr) |  | 0.0254 | -0.0357 – 0.0864 | 0.415 | |
| EQP |  | -0.0243 | -0.0627 – 0.0141 | 0.214 | |
| Smoking habit |  |  |  |  | |
| (current smoker) |  | -0.00991 | -0.0999 – 0.0801 | 0.829 | |
| (ex-smoker) |  | 0.0640 | -0.0664 – 0.194 | 0.335 | |
| Menstrual status |  |  |  |  | |
| (irregular cycles) |  | -0.0367 | -0.116 – 0.0430 | 0.366 | |
| (menopause) |  | 0.0348 | -0.0437 – 0.113 | 0.384 | |
|  |  |  |  |  | |

CI: confidence interval. Analysis of covariance was conducted for log urinary Zn as dependent variable with independent variables (EQP, log daidzein, age and smoking) (N=355). Log daidzein, age, and smoking were included as covariables in addition to EQP in this analysis. BMI was coded as 1= underweight, 2= normal weight, 3= overweight and obese. Equol status was coded as 1= EQP, 2= non-EQP. Tobacco use was coded as 1 = non-smoker, 2 = current smoker, and 3 = ex-smoker. Menstrual status was coded as 1= regular cycles, 2 = irregular cycles, 3 = menopause. Category 1 was set as a reference. Model fitness: R^2^ = 0.0396.

**Table S3.** Association between log urinary Zn and EQP with imputations of missing values in smoking habit and menstrual status.

| **Dependent Variable: Log urinary Zn (μmol/gCr)** | | | | |  |
| --- | --- | --- | --- | --- | --- |
| **Independent variables** |  | *β* | 95% CI | P | |
| BMI |  |  |  |  | |
| (normal weight) |  | 0.0324 | -0.00855 – 0.0734 | 0.121 | |
| (overweight and obese) |  | -0.0121 | -0.0626 – 0.0384 | 0.638 | |
| Age (yr) |  | 0.00262 | -0.00287 – 0.00811 | 0.350 | |
| Log daidzein (μg/gCr) |  | -0.00363 | -0.0572 – 0.0500 | 0.894 | |
| EQP |  | -0.0359 | -0.0700 – -0.00180 | 0.0391 | |
| Smoking habit |  |  |  |  | |
| (current smoker) |  | 0.0154 | -0.0691 – 0.0998 | 0.721 | |
| (ex-smoker) |  | 0.0225 | -0.0982 – 0.143 | 0.714 | |
| Menstrual status |  |  |  |  | |
| (irregular cycles) |  | 0.0543 | -0.0324 – 0.141 | 0.219 | |
| (menopause) |  | 0.0120 | -0.0559 – 0.0798 | 0.729 | |
| (experienced gynecological surgeries) |  | -0.141 | -0.246 – -0.0363 | 0.00837 | |
| Disease histories  (current or past) |  | 0.0228 | -0.0147 – 0.0603 | 0.234 | |
|  |  |  |  |  | |

CI: confidence interval. Analysis of covariance was conducted for log urinary Zn as dependent variable with independent variables (EQP, log daidzein, age and smoking) (N=520). Log daidzein, age, and smoking were included as covariables in addition to EQP in this analysis. BMI was coded as 1= underweight, 2= normal weight, 3= overweight and obese. Equol status was coded as 1= EQP, 2= non-EQP. Tobacco use was coded as 1 = non-smoker, 2 = current smoker, and 3 = ex-smoker. Menstrual status was coded as 1= regular cycles, 2 = irregular cycles, 3 = menopause, and 4 = experienced gynecological surgery. Disease history was coded as 1 = person with current or past disease histories, and 2 = person without disease histories. Category 1 was set as a reference. Model fitness: R^2^ = 0.0355.
